# Supplementary material for: The Dual Prey-Inactivation Strategy of Spiders—In-Depth Venomic Analysis of Cupiennius salei
Source: Toxins (Basel). 2019 Mar 19;11(3):167. doi: 10.3390/toxins11030167 (PMC6468893; doi:10.3390/toxins11030167)
Supplement: Supplementary file 1 [file toxins-11-00167-s001.zip › Supplementary Fig S4/Supplementary Fig S4.pdf]

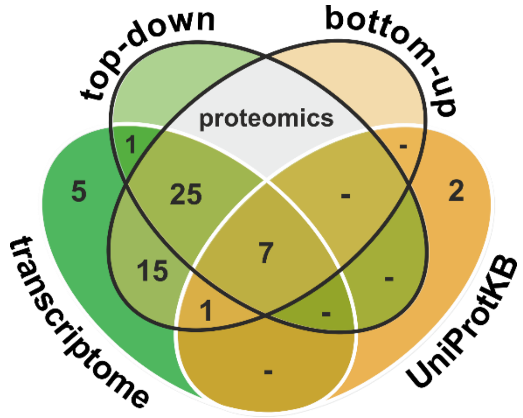

Supplementary Fig S4. Venn diagram of identified (putative) neurotoxins. Only mature peptide isoforms are considered. In our transcriptomic and proteomic study, we could not find sequences corresponding to two peptides formerly sequenced by Edman degradation (UniProtKB: B3EWT1, B3EWT3).
